# Chlorin e6 Phospholipid Delivery System Featuring APN/CD13 Targeting Peptides: Cell Death Pathways, Cell Localization, In Vivo Biodistribution

**DOI:** 10.3390/pharmaceutics14102224

**Published:** 2022-10-18

**Authors:** Yulia A. Tereshkina, Lyubov V. Kostryukova, Elena G. Tikhonova, Yulia Yu. Khudoklinova, Nadezhda A. Orlova, Alisa M. Gisina, Galina E. Morozevich, Pavel A. Melnikov, Vadim S. Pokrovsky

**Affiliations:** 1 Institute of Biomedical Chemistry, 10 Pogodinskaya St., 119121 Moscow, Russia; 2 V. Serbsky National Medical Research Center for Psychiatry and Narcology, V. Serbsky National Medical Research Center for Psychiatry and Narcology, 23, Kropotkinsky Lane, 119034 Moscow, Russia; 3 Federal State Budgetary Institution N.N. Blokhin National Medical Research Center of Oncology of the Ministry of Health of the Russian Federation, 23 Kashirskoe Shosse St., 115478 Moscow, Russia

**Keywords:** phospholipid nanoparticles, targeted drug delivery, tumor, chlorin e6, aminopeptidase N/CD13, cell-penetrating peptide, heptaarginine, colocalization, apoptosis, photosensitizer

## Abstract

We have previously designed a phospholipid delivery system for chlorin e6 to increase the efficacy of photodynamic therapy involving a second-generation photosensitizer. Further research into the matter led to double modification of the obtained nanoparticles with ligands exhibiting targeting and cell-penetrating effects: an NGR-containing peptide and heptaarginine (R7), respectively. This study investigated the cell death pathway on HT-1080 tumor cells after treatment with the proposed compositions: the chlorin e6 phospholipid composition and the two-peptide chlorin e6 phospholipid composition. It was demonstrated that most of the cells died by apoptosis. Colocalization analysis of chlorin e6 in the phospholipid composition with two peptides showed mitochondria are one of the targets of the photosensitizer. An HT-1080 tumor-bearing mouse model was used to evaluate the biodistribution of the drug in tumor, liver, and kidney tissues after administration of the study compositions in comparison with free chlorin e6. The photosensitizer mostly accumulated in the tumor tissue of mice administered the phospholipid compositions, and accumulation was increased 2-fold with the peptide-containing composition and approximately 1.5-fold with the unenhanced composition, as compared with free chlorin e6. The enhancement of the chlorin e6 phospholipid composition with targeting and cell-penetrating peptides was found to be effective both in vitro and in vivo.

## 1. Introduction

Photodynamic therapy (PDT) is a non-invasive treatment of cancer with a minimal risk of toxicity. This method is based on the induction of specific molecules (photosensitizers) producing singlet oxygen and reactive species [1]. The mechanism of PDT depends on reactive oxygen species (ROS) formed in a photochemical reaction and capable of oxidizing large amounts of intracellular active components (such as DNA and lipid compounds) in tumor cells [2]. Chlorin e6 (Ce6), which has a number of useful properties, is one of the well-known and widely used photosensitizers (PSs) [3,4,5]. However, second-generation PDT agents, including Ce6, have several crucial limitations: poor water solubility resulting in unfavorable pharmacokinetics, long-term phototoxicity, low efficacy, and limited depth of tumor exposure [6]. When exposed to the light of a certain wavelength, PSs release singlet oxygen (^1^O_2_), which can kill cancer cells. However, due to the short half-life (<40 ns) of ^1^O_2_ during PDT, each ^1^O_2_ molecule can exert a therapeutic effect only within an area with a diameter of less than 20 nm [7,8]. Therefore, for the proper action of a PS, it has to be delivered right into the tumor cell, which will avoid potential cytotoxicity in normal tissues [4].

One approach to address these drawbacks is to use delivery systems based on various carriers for PS, including liposomes, micelles, polymer nanoparticles, and inorganic nanoparticles, in order to increase the accumulation and retention of PSs in tumor tissue and their absorption by tumor cells [9]. On the other hand, nanoparticles decorated with specific monoclonal antibodies or ligands have been used as carriers allowing tumor-targeting drugs to access and detect tumor cells that express specific antigens or cell surface receptors [10]. Targeting peptides can act as such ligands [11,12,13]. In recent decades, particular interest has been paid to asparagine–glycine–arginine (NGR) peptides, which can be used for ligand-targeted delivery of various chemotherapeutic drugs to the vascular network of a tumor. The key binding site of NGR peptides in the tumor vascular network is aminopeptidase N (APN; CD13) [14]. APN/CD13 is a weighted glycosylated zinc-dependent transmembrane ectopeptidase with a molecular weight of 160 kDa. It has three main functions, acting as an enzyme, a receptor, and a signaling molecule. In addition, APN/CD13 participates in the degradation of extracellular matrix proteins, which promotes tumor cell invasion and migration. Studies have reported that APN/CD13 is overexpressed in endothelial cells of the tumor vascular network and in some solid tumors (melanoma, prostate carcinoma, lung cancer, pancreatic adenocarcinoma, ovarian cancer, breast cancer, colon cancer, etc.). Because of the increased expression, APN/CD13 can be regarded not only as an important clinical marker in some inflammatory diseases and malignancies, but also as a suitable target for antitumor and anti-inflammatory therapy [15]. The accessible location of the target (i.e., the tumor vascular network) and the high specificity of the ligand make NGR a suitable candidate for active targeting of macromolecule or nanoparticle systems in vivo. In addition, preclinical evaluation of liposomes containing NGR and doxorubicin showed that conjugation of NGR-TNF α1-11 with the liposome surface leads to a significant improvement in tumor accumulation and efficacy compared to the untargeted composition [16].

**Scheme 1:**
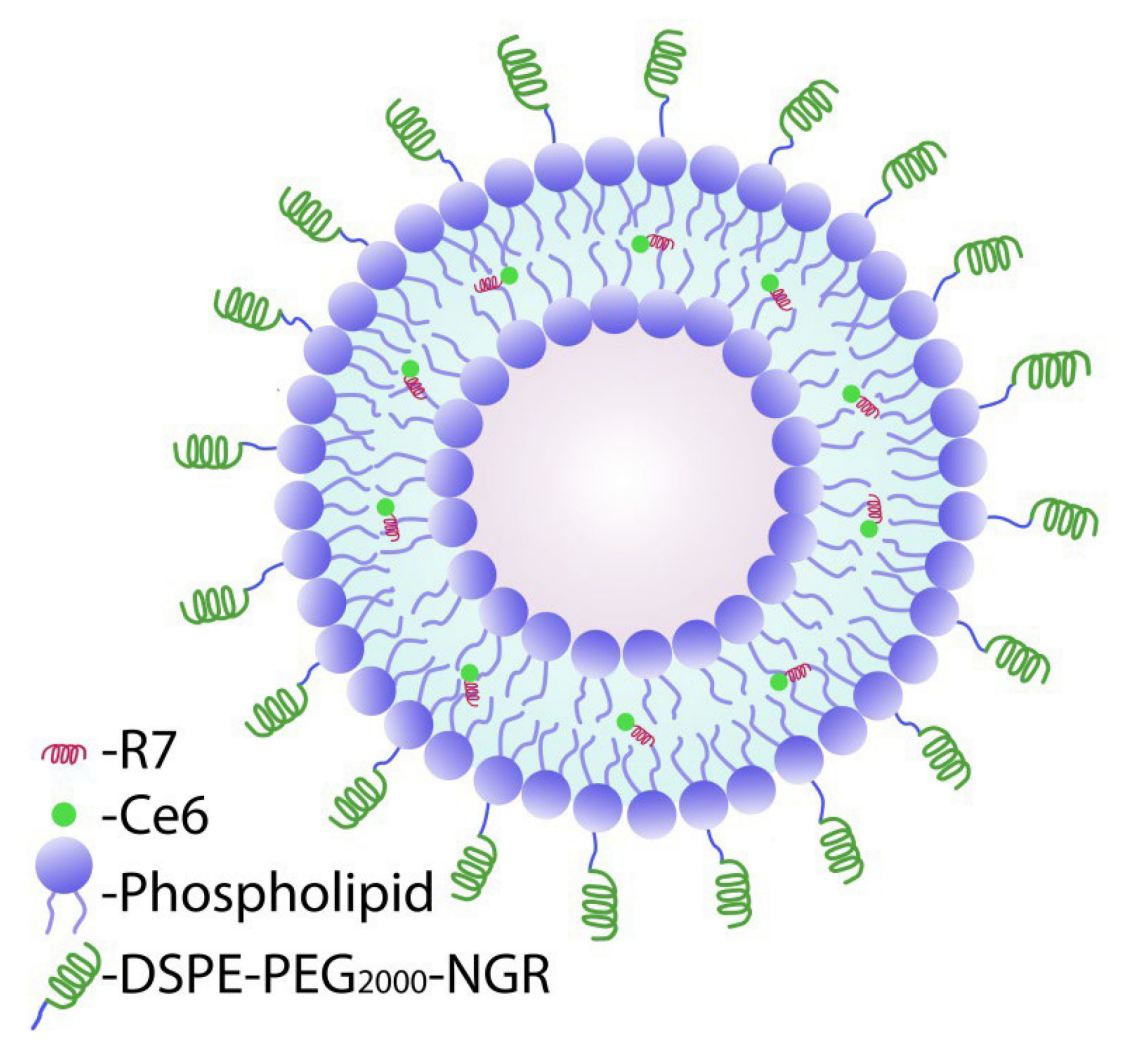
Model of the Ce6 incorporated to phospholipid system with peptides.

In addition to targeting peptides, a number of cell-penetrating peptides are used in studies to increase cell penetration [17,18,19,20]. Such peptides may include oligoarginines, in particular heptaarginine (R7). The internalization of such peptides is associated with interaction with the cell membrane [20]. Most cell-penetrating peptides have a positive charge (83%) due to the presence of a large number of basic residues (arginine or lysine) in their sequence. The overall positive charge promotes interaction with negatively charged membranes, which allows their cellular internalization [21]; however, the mechanism of cellular absorption has not yet been revealed [22,23]. In our previous in vitro experiments [24], we showed that the use of a peptide containing an NGR and a cell-penetrating peptide (R7) enhanced the effect of the chlorin e6 phospholipid composition. Additional in vivo studies were conducted to further understand the mechanism of action and behavior of the designed Ce6 composition with a targeting and a cell-penetrating peptide (a variant of the possible type of the phospholipid system Ce6 with peptides developed by us is shown in Scheme 1).

We believe that the modification of the previously developed Ce6 phospholipid composition with two peptides should provide better efficacy of the PS itself. It was of particular interest to investigate the PS, which localizes in cellular organelles/compartments depending on the type of its delivery into cells (in free form, in nanoparticles with and without peptides), the cell death pathway, as well as the accumulation of Ce6 in the tumor tissue and in some excretory organs (kidneys, liver).

## 2. Materials and Methods

### 2.1. Materials

The soy phospholipid Lipoid S100 with a phosphatidylcholine content of 94–96% was purchased from Lipoid S100 (“Lipoid”, Ludwigshafen, Germany); maltose monohydrate was purchased from Merck Merck KGaA, Darmstadt, Germany); DSPE-PEG(2000)-Maleimide was purchased from Avanti (Avanti, Alabaster, AL, USA); the drug substance di-N-methyl-D-glucamine salt of chlorin e6, with a 50% content of chlorin e6 was purchased from the State Educational Institution of Higher Professional Education “Ivanovo State University of Chemistry and Technology” (ISUCT, Ivanovo, Russia). A specific peptide with the NGR-motif (NH2-)Gly-Asn-Gly-Arg-Gly-Cys(-COOH) with a purity of not less than 95% was obtained from Sinton-Lab LLC (Sinton-Lab LLC, Saint Petersburg, Russia). The penetrating peptide, heptaarginine (R7), was synthesized at the Institute of Biomedical Chemistry (CCU Human Proteome, IBMC, Moscow, Russia) by solid-phase peptide synthesis using a 433A synthesizer (Applied Biosystems, Waltham, MA, USA).

High-purity solvents and chromatography-grade reagents were used: methanol HPLC grade (Fisher Scientific UK Ltd., Altrincham, UK); acetonitrile HPCL, far UV (Acros Organic, Fair Lawn, NJ, USA); formic acid 98–100% (Merck KGaA, Darmstadt, Germany); trifluoroacetic acid, for protein sequence analysis ≥99.5% (Fluka Chemie AG, Buchs, Switzerland), water purified using a Milli-Q Biocel A10 apparatus (Milli-Q, Darmstadt, Germany) (purified water) or distilled water (GFL-2004 water distillation unit manufactured by GFL).

Cell cultures were incubated using RPMI-1640 culture media (PanEco, Moscow, Russia), Versene solution (PanEco, Moscow, Russia), and fetal bovine serum (FBS) (Gibco, Gaithersburg, ML, USA).

### 2.2. Preparation of Chlorin e6 Formulations

Peptide-containing Ce6 formulations were prepared according to the previously described procedure [24]. Phospholipid nanoparticles containing Ce6 (NPh-Ce6) were obtained by homogenizing a coarse aqueous emulsion of Ce6 and phospholipid (mass ratio 1:10) using a Bandelin Sonopuls ultrasonic homogenizer (Bandelin, Dusseldorf, Germany) (KE72 rod, for 6 min at 50% power). To obtain a two-peptide composition (NPh-Ce6-NGR-R7), the targeting peptide (with the NGR motif) was first built into the NPH-Ce6 composition by the film method. DSPE-PEG2000 conjugate with maleimide was obtained using the molar ratio of the main components of DSPE-PEG2000-Mal: NGR of 4.3:1. Lipoid S100 was dissolved in 96% ethanol (Medkhimprom, Roshal, Russia) at a mass ratio of 125:1; separately, DSPE-PEG2000-NGR conjugate was dissolved in 96% ethanol at a ratio of 25:1 (*w*/*w*). The obtained alcohol solutions were mixed at a lipid-to-conjugate ratio of 10:1 (*v*/*v*). Alcohol was removed using a Heidolph Laborota 4003 rotary evaporator (Heidolph, Schwabach, Germany) until a dry film was obtained. The resulting film was rehydrated with an aqueous solution of Ce6 (Ce6 to lipid mass ratio 1:10). The emulsion was treated with an ultrasonic disintegrator under the same conditions as NPh-Ce6 (described above). After that, peptide R7 was added to the resulting emulsion (peptide R7 to Ce6 ratio 6:1) and intensively shaken on an IKA MS 3 basic shaker (IKA, Canton, MA, USA). All obtained ultra-thin emulsions were passed through a 220 nm filter (Merck KGaA, Darmstadt, Germany). In all samples, the particle size was less than 40 nm, with more than 96% of the Ce6 amount included in phospholipid nanoparticles.

### 2.3. Cell Line

The study used a human fibrosarcoma cell line (HT-1080 cell culture) obtained at the D. I. Ivanovskiy Institute of Virology (Moscow, Russia). Tumor cells were cultured according to the recommendations in the cell culture certificate using appropriate media fortified with 10% FBS [25]. The cells were cultured at 37 °C in a humid atmosphere with 5% CO_2_ (Binder CO_2_ Incubator, Binder, Tuttlingen, Germany). Cell lines were used after 3 to 10 passages.

### 2.4. Evaluation of CD13 Expression in Tumor Samples and Cell Lines by Western Blot

CD13 expression in HT-1080 tumor cells and the HT-1080 xenograft tumor was determined by Western blot. The tumor was homogenized using a manual pestle homogenizer (Duran, Wertheim, Germany). The homogenates were centrifuged (10,000 rpm, 10 min), and the supernatant was collected. Protein was determined in the tumor homogenates and cell lysates in the Bradford assay. Tumor and tumor cell lysates (50 µg protein) were subjected to electrophoresis in 7.5% polyacrylamide gel (PAGE) (Bio-Rad, Hercules, CA, USA) in SDS running buffer (Bio-Rad, USA). After electrophoresis, the proteins were transferred to nitrocellulose membranes and blotted with the primary antibody to rabbit mAb CD13/APN (D6V1W) (Cell Signaling, Danvers, MA, USA). Then, they were washed and stained with a secondary anti-rabbit antibody (Amersham, Little Chalfont, UK). The bands were developed in an ECL detection system (Bio-Rad, USA) and imaged using a ChemiDoc system (Bio-Rad, USA).

### 2.5. Confocal Laser Scanning Microscopy

The cells were seeded into confocal dishes (density 5.0 × 10^4^ cells/well) and cultured for 24 h. After that, the cells were incubated with Ce6, NPh-Ce6, and NPh-Ce6-NGR-R7 (chlorin e6 concentration 5 µM) for 30 min. Then, the Ce6 phospholipid composition samples were removed and washed twice with DMEM. Afterward, LysoTracker™ green DND-26 was added to the dishes, which were incubated in DMEM for 10 min. Then MitoTracker™ Red CMXRos (without washing) was introduced into the dishes, followed by 10 min incubation. After that, the dishes were placed on a confocal microscope with a CO_2_ incubator (37 °C, 5% CO_2_) for intravital microscopy (Tokai Hit, Fujinomiya-shi, Japan). Scanning was performed using a Nikon A1R MP+ laser scanning confocal microscope (Nikon, Tokio, Japan). Lasers with emission wavelengths of 488 nm (Em 500–550 nm), 561 nm (Em 570–620 nm), and 638 nm (Em 663–738 nm) were used in this study.

The optic tools included Plan Apo 20x/0,75 Dic N, Apo IR 60x/1,27 WI, and Apo TIRF 60x/1,49 oil Dic lenses (Nikon, Japan). Cell contours were visualized using differential interference contrast. Neural network processing and binarization of the obtained images were perfor med using NIS-Elements AR software (Nikon, Tokio, Japan). Colocalization with subcellular compartments was performed using the Pearson correlation coefficient (PCC) and the Manders overlap coefficient (MOC) in NIS-Elements AR software (Nikon, Tokio, Japan).

### 2.6. Apoptosis Analysis

Apoptosis in HT-1080 cells incubated with Ce6 composition samples after PDT was analyzed using a special kit, FITC Annexin V Apoptosis Detection Kit I (BD Biosciences, San Jose, CA, USA). The cells were seeded in 6-well plates (density of 1 × 106 cells/mL). After attachment, the cells were incubated with aqueous solutions of chlorin e6 (Ce6, NPh-Ce6, and NPh-Ce6-NGR-R7) samples with a concentration of 2.0 µg/mL for 2 h. Next, the cells were irradiated with a 638 nm laser (experimental laser apparatus, Institute of Photonic Technologies, Federal Research Center for Crystallography and Photonics, Russian Academy of Sciences, Moscow, Russia) for 20 min (1 J/cm^2^ dose). After that, the Ce6 samples were removed and the cells were washed twice with PBS. Fresh DMEM medium was poured into the cells, which were then left in a CO_2_ incubator (37 °C, 5% CO_2_) for 24 h. Afterward, the cells were treated with a Trypsin/Versene solution (1:1), washed in PBS, and resuspended in 100 µL of Annexin V binding buffer. The cells were incubated with 5 µL of Annexin V-FITC and 5 µL of PI in the dark for 15 min at room temperature. A FACSAria III cell sorter/flow cytometer (BD Biosciences, San Jose, CA, USA) equipped with a blue (488 nm) and a yellow-green (561 nm) laser was used for the cell staining analysis. The results were analyzed using BD FACSDiva Software Version 7. A graphical representation of data was prepared using FlowJ version 10 software.

### 2.7. Animal Model

Female Balb/c nude animals (age 4–6 weeks, body weight 20–25 g) were obtained from the Federal State Budgetary Institution “N. N. Blokhin National Medical Research Center of Oncology” of the Ministry of Health of Russia (NMRC of Oncology) (Moscow, Russia). Before the experiment, the mice were kept for 14 days under standard conditions (12 h of light, temperature 20 °C). The animals received dry chow and water ad libitum. After in vitro culture and testing for CD13 expression, HT-1080 tumor cells suspended in 50 µL of the cell culture medium and 50 µL of BD Matrigel (BD Biosciences, San Jose, CA, USA) were administered to the mice by subcutaneous injection into the flank of the body (3 × 10^6^ cells/mouse). Mice with the inoculated tumor were kept for 2 weeks until the tumor volume reached 200–300 mm^3^. Tumor growth was measured in three orthogonal measurements using a caliper. All animal studies were performed according to the European Convention for the Protection of Vertebrate Animals used for Experimental and Other Scientific Purposes. Animal protocols were approved by the local ethics committee of the N. N. Blokhin National Medical Research Center of Oncology (Decision 2021-17 issued on 5 October 2021).

### 2.8. In Vivo Biodistribution

Mice with developed xenografts were administered free Ce6, NPh-Ce6, and NPh-Ce6-NGR-R7 at a dose of 5 mg/kg into the tail vein. At certain time points, three animals from each group were euthanized by cervical dislocation. The tumor and organs (liver and kidneys) were excised and analyzed by mass spectrometry (LC-MS) using an Agilent 1200 Series liquid chromatograph and a Quadrupole LC/MS 6130 mass spectrometric detector manufactured by Agilent Technologies (USA). Extraction of chlorin e6 from the tumor and organs (liver and kidneys), as well as mass spectrometry analysis, was performed as previously described in our publication [26].

The analysis was performed using selected ion monitoring in positive mode. Ce6 was registered at *m*/*z* of 597.7. The mobile phase consisted of acetonitrile with 0.1% formic acid (B)/water with 0.1% formic acid (A). The sample was injected onto a 4.6 × 150 mm (5 μm) Eclipse XDB-C18 column (Agilent Technologies, Santa Clara, CA, USA) and eluted with a mobile phase consisting of acetonitrile with 0.1% FA (B)/water with 0.1% FA (A). The composition of the mobile phase was changed as follows, time in min (% B): 0(70)-3(90)-10(90) and subsequently equilibrated at 70% B for 3 min, resulting in a total run time of 13 min. Calibration dependence of the ratio of peak areas was linear in the Ce6 concentration range from 0.1 to 10 μg·mL^−1^. The correlation coefficient was 0.9995. Chromatograms were treated using ChemStation B.01.03 software.

### 2.9. Statistical Analysis

The statistical significance of the data was analyzed by the independent samples *t*-test using SPSS Statistics R24.0 software (IBM). Data in the figures and tables are presented as mean ± standard error of the mean. *p*-values < 0.05 were considered statistically significant (* *p <* 0.05).

## 3. Results

### 3.1. Intracellular Localization of Chlorin e6 In Vitro

**Figure 1:**
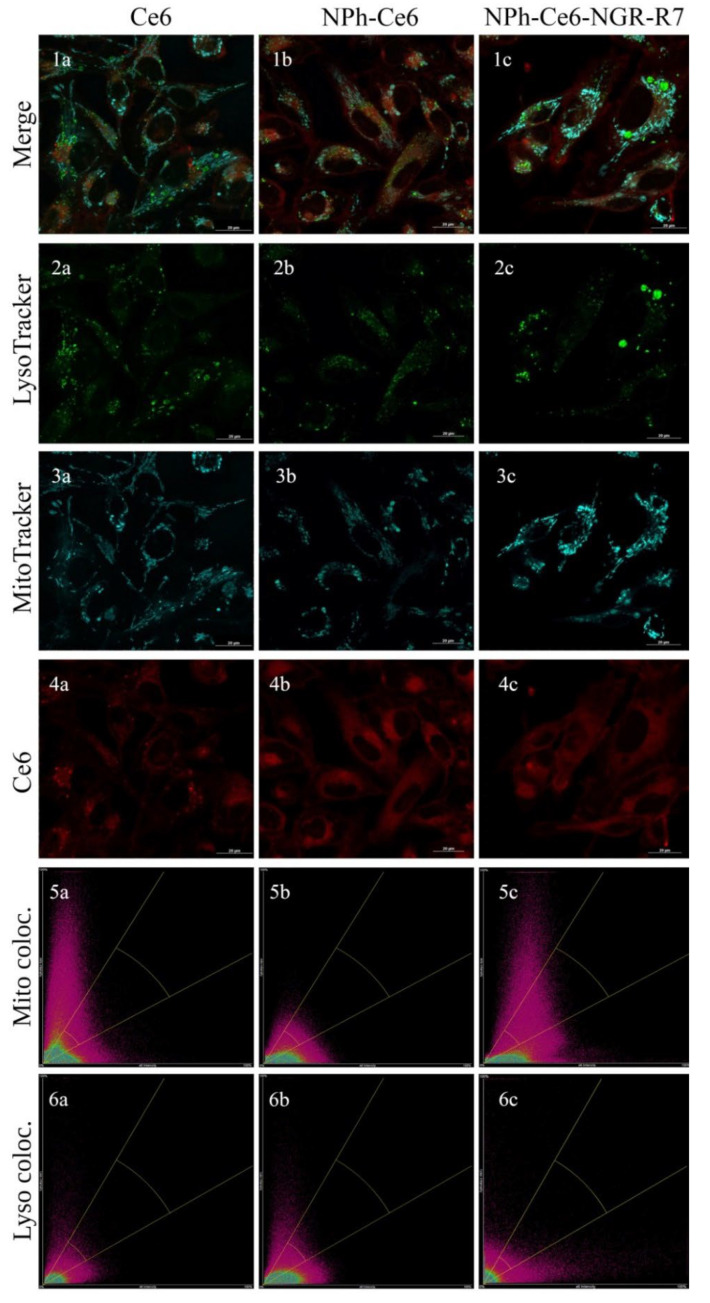
Colocalization analysis of chlorin e6 in the HT-1080 cell line, where: (**a**) Ce6, (**b**) NPh-Ce6, (**c**) NPh-Ce6-NGR-R7 and corresponding channels (**1**) Merge image, (**2**) LysoTrackerTM Green DND-26, (**3**) MitoTracker™ Red CMXRos, (**4**) chlorin e6 compositions, (**5**) Mito colocalization, (**6**) Lyso colocalization. Scale—20 µm.

A key aspect of the interaction of a PS with the cells of a target tissue or tumor is the subcellular localization of the PS. A PS can localize in the mitochondria, lysosomes, endoplasmic reticulum, Golgi apparatus and plasma membrane, and cytoskeleton [2]. A PS entry into the tissue triggers an acute stress response leading to changes in the metabolism of calcium and lipids, and the production of cytokines and stress proteins. The majority of cellular reactions are known to occur in mitochondria [27]. The determination of molecular targets for a PS is a complex but important study that can help to optimize the PDT process and choose a rational treatment method. This study is especially important for newly developed delivery systems, since the added components can affect the intracellular localization of the PS. Using confocal microscopy, we investigated the dependence of the effect of the delivery system used for chlorin e6 on its intracellular localization (Figure 1).

It has been demonstrated that 40 min exposure of Ce6 in a cell-containing medium is sufficient for its accumulation by HT-1080 cells. A 40 min exposure of Ce6 could be detected in subcellular compartments. As a result, chlorin e6 from the NPH-Ce6-NGR-R7 sample was partially colocalized in mitochondria (Figure 1). Analysis of this Ce6 composition with the peptides showed an unreliable match of channels with the lysosomal tracker PCC = 0.37, MOC = 0.52, and a reliable match of channels with the mitochondrial tracker PCC = 0.51, MOC = 0.60, indicating mitochondrial localization. Analysis of NPH-Ce6 colocalization with the lysosomal tracker shows PCC = 0.22, MOC = 0.57, which indicates an unreliable match of channels. Analysis of NPH-Ce6 colocalization with the mitochondrial tracker reveals PCC = 0.37, MOC = 0.64, which indicates a partial overlap of channels, and therefore mitochondrial localization of the nanoparticles (NPs). The colocalization analysis of free chlorin demonstrated PCC = 0.39, MOC = 0.65 for the lysosomal tracker and PCC = 0.33, MOC = 0.52 for the mitochondrial tracker, which, in both cases, indicated no channel match. It may be located in other compartments. A photo of the 3D reconstruction of the cells depending on the delivery system used is presented in the Supplementary Section.

Thus, mitochondria were shown to be one of the final targets for the NPh-Ce6-NGR-R7 composition (phospholipid nanoparticles with embedded Ce6, and a cell-penetrating and targeting peptide) in the HT-1080 cell culture. Free Ce6 substance and phospholipid nanoparticles with embedded Ce6 without the peptides had less affinity for these organelles and could not be statistically confirmed; although, in fact, this is not the case; we detected rare events of matching of other forms of chlorines with Mitotracker, which suggests that the modification of chlorin did not radically change the selectivity of the NP uptake. These results may indicate an influence of other components of the Ce6 delivery system on their intracellular localization (possibly due to interactions of lipids with cell organelles).

### 3.2. Induction of Apoptosis in HT-1080 Cell Line

**Figure 2:**
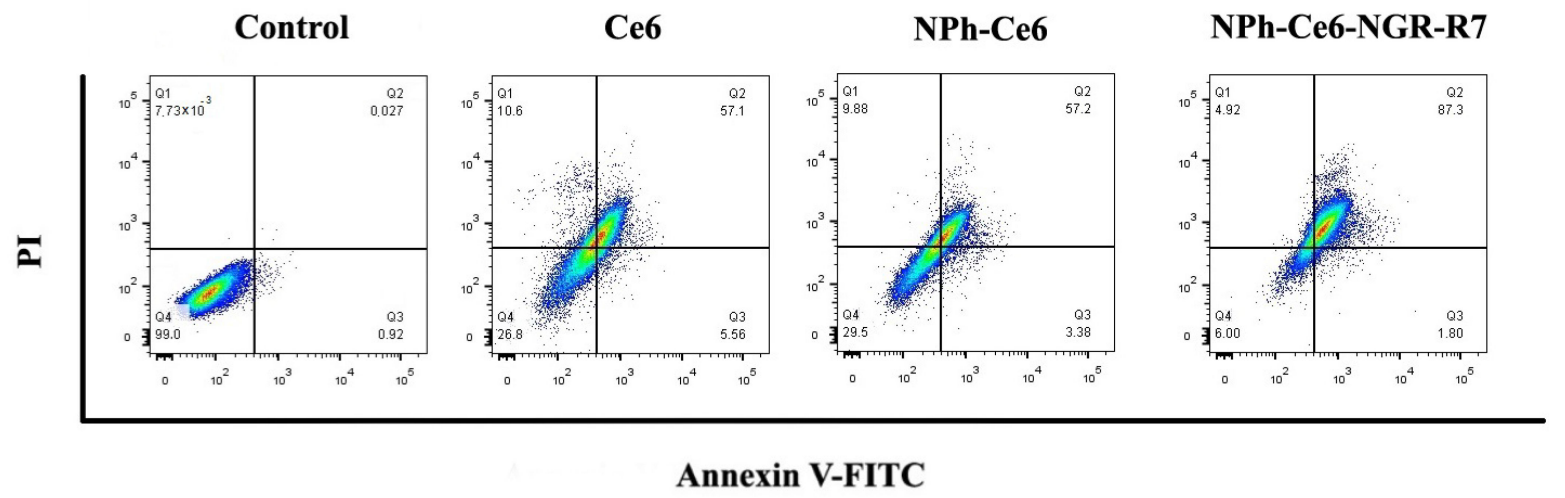
Analysis of apoptosis in HT-1080 cell culture after PDT (e6 chlorine concentration 2.0 mcg/mL, irradiation dose 1 J/cm^2^) for cells incubated with free Ce6; phospholipid composition NPh-Ce6; phospholipid composition with peptides NPh-Ce6-NGR-R7. Quadrant design: top left (Q1)—necrosis, cells stained with PI; top right (Q2)—late apoptosis; cells stained with PI and Annexin V simultaneously; bottom right (Q3): early apoptosis, cells stained with Annexin V; bottom left (Q4)—fluorescent signal at the level of autofluorescence of unstained cells.

The entry of a PS into the tumor tissue is followed by an acute stress response changing calcium and lipid metabolism, the production of cytokines and stress proteins, activation of enzymes (in particular, protein kinases), and expression of transcription factors. These effects often induce apoptosis in the mitochondrial pathway that includes caspases through the release of cytochrome c or by pathways involving ceramides or death receptors. However, under certain circumstances, PDT-exposed cells also die as a result of necrosis [27]. Since no studies of tumor cell death pathways had been conducted with the proposed Ce6 phospholipid delivery systems, we analyzed the degree of apoptosis and necrosis in the HT-1080 cell line after irradiation using flow cytometry. The cells were stained with Annexin V-FITC to determine apoptotic cells, and the fluorescent dye propidium iodide (PI) was used to determine dead cells. The results of this analysis are presented in Figure 2 as dot plots divided into four quadrants.

The study of the effect of e6 chlorine compositions after PDT on the death of fibrosarcoma tumor cells (HT-1080) using flow cytometry revealed that 24 h after treatment most cells were in late-phase apoptosis (Figure 2). The percentage of cells in a state of necrosis (Q1) for all the substances studied did not exceed 11%. The pattern of late apoptosis (Q2) was observed in 57.1%; 57.2%, and 87.3% of cells for Ce6, NPh-Ce6, and NPh-Ce6-GR-R7, respectively. The percentage of cells in early-phase apoptosis for all the drugs studied was no more than 6%. Thus, it was shown that treatment of Ce6 both in its free form and as part of nanoparticles in combination with PDT led to cell death mainly by apoptosis.

### 3.3. In Vivo Efficacy of Chlorin e6

An important aspect in the treatment of cancer is the extent to which PDT damages tumor and normal cells. The expression of oncogenes in cancer cells can affect susceptibility to PDT, while tumor cell selectivity can be increased by equipping the PS with a delivery system containing a targeting component—in this case, an NGR peptide specific to aminopeptidase N (APN; CD13).

**Figure 3:**
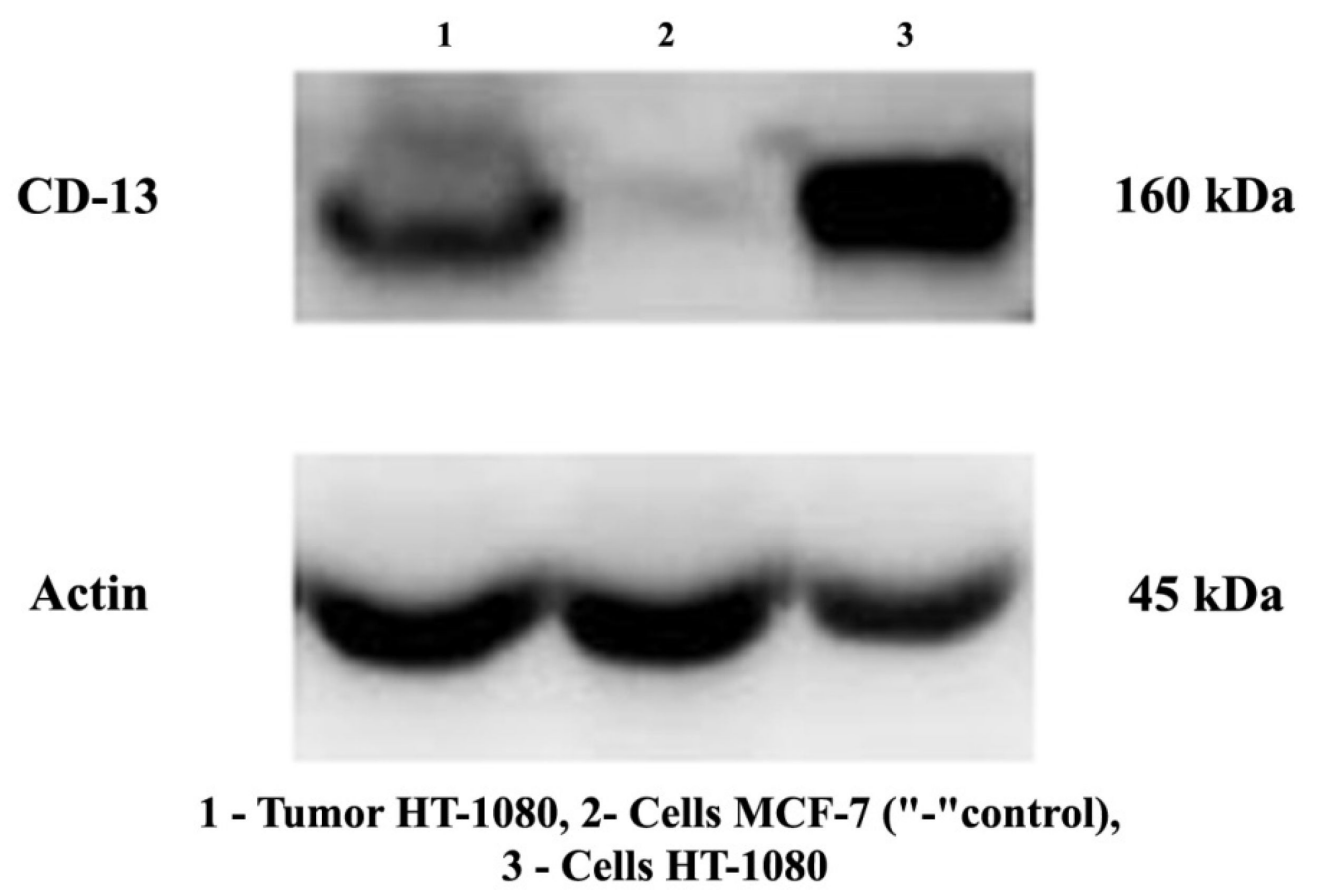
CD13 expression in HT-1080 xenograft tumor, HT-1080 and MCF-7 cell lines (negative control) by Western blot.

Prior to the in vivo studies, the expression of CD13 protein in both the inoculated tumor and the HT-1080 cell line was assessed by Western blot. The results revealed an expression of this protein on the surface of tumor cells in the study samples (Figure 3).

**Figure 4:**
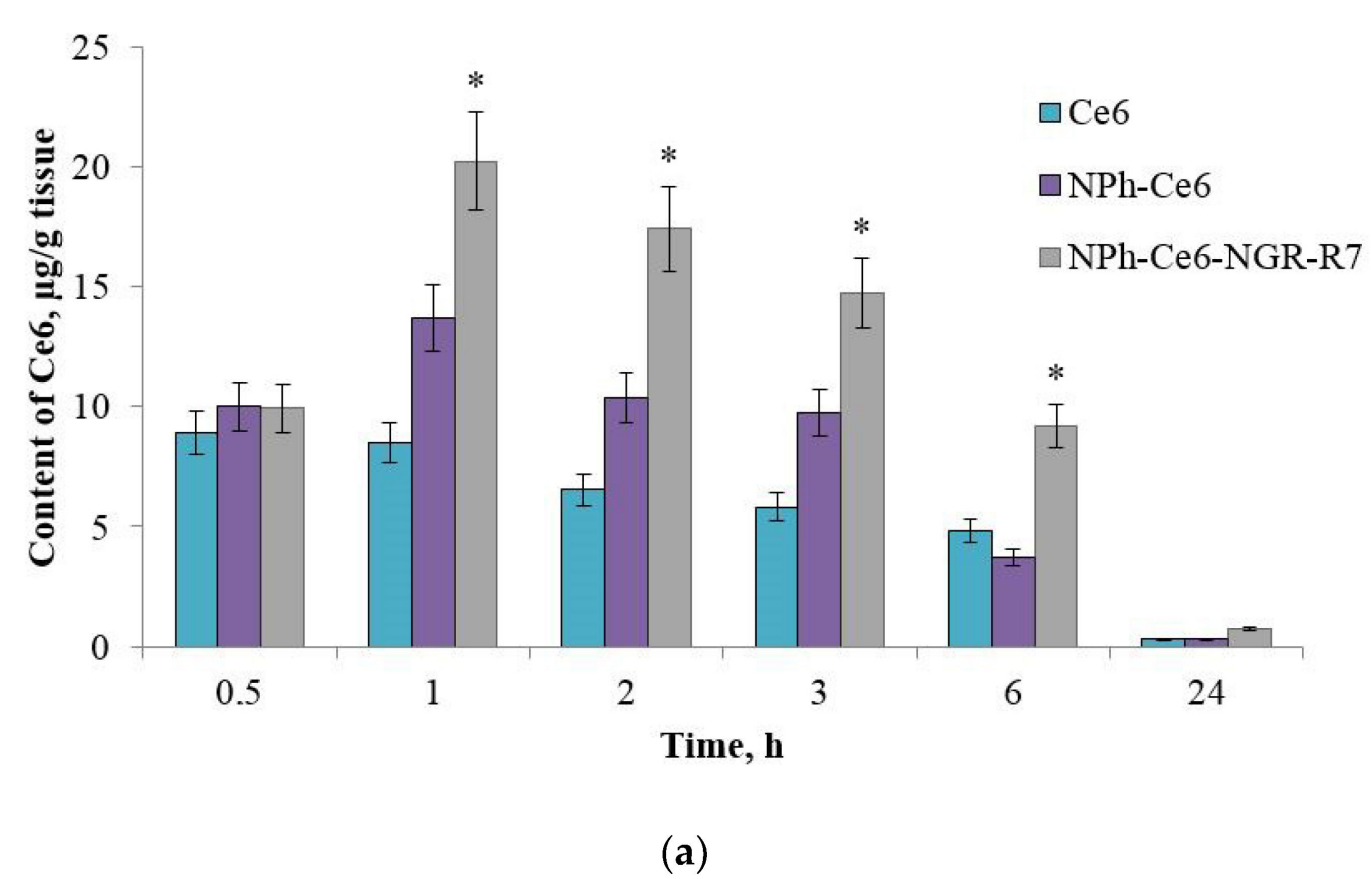

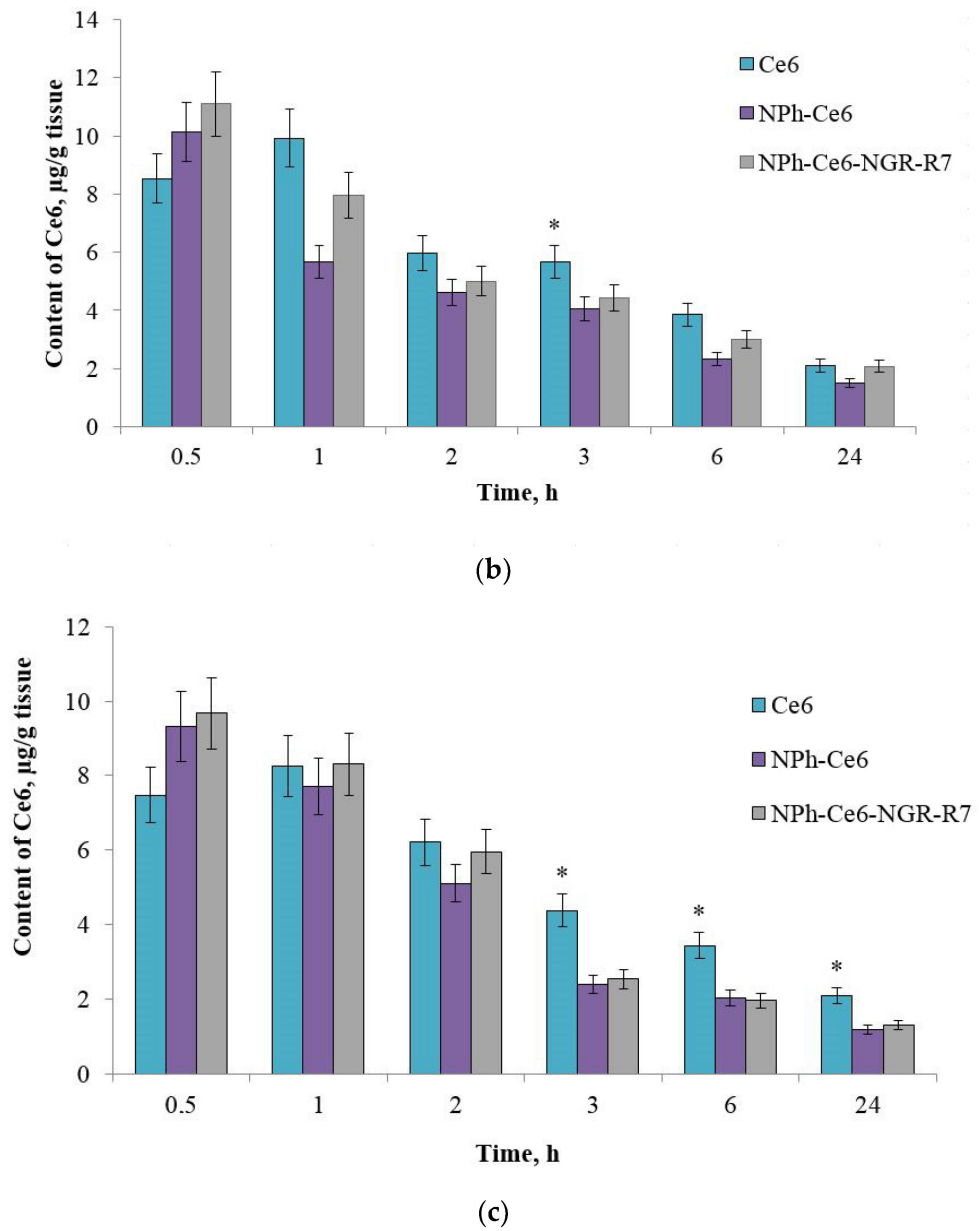
The content of chlorin e6 in tumor tissues (**a**), liver (**b**), and kidneys (**c**) of HT-1080 mice following intravenous administration of the chlorin e6 compositions: free Ce6 substance (blue line), NPh-Ce6 (purple line), NPh-Ce6-NGR-R7 (black dotted line). The dose of Ce6 is 5 mg/kg. * *p* < 0.05 (*n* = 3).

After confirming the CD13 expression of the HT-1080 xenograft, the mice were administered compositions with the PS (free Ce6, phospholipid composition NPh-Ce6, and peptide-enhanced phospholipid composition NPh-Ce6-NGR-R7). Accumulation assessment revealed that the use of peptides as a targeting vector increased the accumulation of Ce6 in the tumor almost 1.5-fold compared to free Ce6 (Figure 4a).

The maximum accumulation occurred 1 h after the administration (20.5 µg/g of tumor mass), followed by a gradual decrease in the tumor PS level and trace amounts at 24 h. The differences were statistically significant, *p* < 0.05 (*n* = 3).

Evaluation of Ce6 accumulation in tissues with the highest blood supply and involved in the elimination of the drug, the liver (Figure 4b) and kidneys (Figure 4c), showed that the maximum accumulation of Ce6 was achieved 30 min after administration.

After administration using the phospholipid delivery systems NPh-Ce6-NGR-R7 and NPh-Ce6, the concentrations were 11 µg/g of liver tissue and 10.3 µg/g of liver tissue, respectively, and 9.5 µg/g of kidney tissue and 9.1 µg/g of kidney tissue, respectively. For free Ce6 substance, the maximum values were obtained at 1 h, being 10 µg/g of liver tissue and 8.6 µg/g of kidney tissue. At 24 h, the Ce6 levels in the liver were almost the same for NPh-Ce6-NGR-R7 and free substance. In the kidneys, the 24 h content was almost two times higher with free Ce6 than with embedded Ce6 (NPh-Ce6-NGR-R7 and NPh-Ce6).

Thus, the in vivo study revealed that the accumulation of Ce6 in the tumor tissue was twice that in the liver and kidneys 1 h after administration using phospholipid NP with peptides. At the same time, the Ce6 level was 1.5 times higher with phospholipid NP without the peptides than with free substance 1 h after the PS was administered. This means that the use of a targeting and a cell-penetrating peptide increased the accumulation of the drug in the tumor target.

## 4. Discussion

In our previous papers [24,26], we reported on the preparation of a Ce6 phospholipid composition and its additional modification with two peptides with cell-penetrating (an arginine-containing peptide R7) and targeted actions (a peptide containing the NGR motif). Aminopeptidase N (APN) located on the surface of tumor cells was selected as a pharmacological target for the developed composition [28]. We characterized the physicochemical parameters of the composition of Ce6 embedded in phospholipid nanoparticles (NPh-Ce6) and this composition with two peptides (NPh-Ce6-NGR-R7). These compositions were ultra-thin emulsions with <40 nm particles. The peptides increased both the accumulation of Ce6 inside the cell and its photoinduced action. However, we have not previously studied the pathways and mechanisms of penetration of the Ce6 phospholipid composition into the cell; therefore, this research comprises additional studies conducted to understand the possible mechanisms of this action.

A key aspect of the interaction of a PS with the cells of a target tissue or tumor is its subcellular localization. A study of the PS distribution in cellular organelles is particularly important for newly developed delivery systems, since each component added can affect the intracellular localization of the PS. A PS can localize in the mitochondria, lysosomes, endoplasmic reticulum, Golgi apparatus and plasma membrane, and cytoskeleton [5]. A PS entry into the tumor tissue triggers an acute stress response leading to changes in the metabolism of calcium and lipids, and the production of cytokines and stress proteins [27]. The determination of molecular targets for PS can help to optimize the PDT process and choose a rational treatment method. Using confocal microscopy, we showed that the compositions of Ce6 without peptides and free Ce6 had a lower affinity for mitochondria than the composition of Ce6 with peptides. It is possible that the presence of the CPP (R7) contributed to a more intensive passage of particles Ce6-carrying through the mitochondrial membrane. In lysosomes, there was no reliable match of channels for both phospholipid compositions, regardless of the presence of peptides. No reliable match of channels was observed for free Ce6 with either the mitochondrial or lysosomal tracker, which may be due to the PS localization in other cellular compartments. The localization of Ce6 in mitochondria was studied in [29], which reports that liposomal Ce6 is predominantly localized in the endoplasmic reticulum, Golgi apparatus, and cell mitochondria, while its levels in lysosomes are low. In contrast, an in vitro study [30] carried out with glucose-conjugated Ce6 has shown that the PS is mainly localized in lysosomes.

There is evidence that the accumulation of photosensitizers in cell organelles also depends on the PS charge: for example, cationic compounds accumulate predominantly in mitochondria, while anionic compounds, in lysosomes [5]. Our previous results confirm this fact, namely, the zeta potential of the Ce6 phospholipid compositions with and without two peptides was 6.21 ± 2.0 mV (accumulation of PS in mitochondria was observed) and −23.3 ± 6.6 mV, respectively [24]. Although, in the latter case, we do not exclude the PS distribution to other cellular compartments, which requires further investigation.

As for the predominant localization of the developed composition of Ce6 with two peptides in mitochondria, this is most desirable. Due to the very short life (~40 ns) and diffusion radius (~20 nm) [31], PDT-induced cytotoxic ROS must be generated near targeted organelles or cellular compartments in order to be effective. Therefore, the majority of studies currently focus on the development and production of mitochondria-targeted delivery systems for PDT agents, and in this regard, arginine-rich cell-penetrating peptides are of particular importance. In particular, the described liposomes based on second-generation arginine-rich dendritic lipopeptides demonstrated the best efficacy in targeting mitochondria [32]. In our case, the double modification of the chlorin e6 phospholipid system with targeting and cell-penetrating peptides also ensured delivery of the PS to mitochondria, facilitating its passage through various physiological and biological barriers. Previously, we showed that the addition of an NGR-containing peptide and R7 to the Ce6 phospholipid nanosystem increased the accumulation of this PS in HepG2 cells expressing aminopeptidase N [24]. We confirmed the presence of this receptor protein on the surface of the studied HT-1080 cells by Western blot (Figure 3), which was also confirmed by other authors [33]. The NGR-containing peptide and R7 may play a crucial role in this process at the initial stage of the PS passage into the cell, contributing to the process acceleration. However, the authors of [30] argued that intracellular localization did not depend on the composition of the Ce6 derivatives they investigated, while the pharmacological composition strongly affected the cellular uptake and accumulation kinetics of the drug.

The entry of a PS into the tumor tissue is followed by an acute stress response changing calcium and lipid metabolism, the production of cytokines and stress proteins, activation of enzymes (in particular, protein kinases), and expression of transcription factors. These effects often induce apoptosis in the mitochondrial pathway that includes caspases, through the release of cytochrome c or via pathways involving ceramides or death receptors. However, under certain circumstances, PDT-exposed cells also die as a result of necrosis [27]. Our analysis of a study of the degree of apoptosis and necrosis in the HT-1080 cell line after irradiation using flow cytometry showed that tumor cells treated with Ce6 phospholipid compositions (NPh-Ce6 and NPh-Ce6-NGR-R7) and free Ce6 died mainly by apoptosis. It can be assumed that PSs exert their damaging effect either by directly damaging DNA, or through another possible mechanism involving caspases (mitochondrial pathway) or the release of cytochrome c or a ceramide, or death receptors. Presumably, in the case of the two-peptide Ce6 phospholipid composition, apoptosis most likely occurred via the mitochondrial pathway, which is confirmed by the results of our PS colocalization analysis. Some authors report that the photosensitization of mitochondria activates apoptotic cell death pathways [29,34,35,36]. On the other hand, it is reported in [37] that Light-activated Photolon, a derivative of chlorine, caused no increase in caspase-3/7 activity, but increased the release of lactate dehydrogenase, causing cell death by necrosis.

Despite PDT being considered the most sparing, non-invasive treatment method for cancer, there are certain difficulties with the use of PS, especially their non-selective accumulation in healthy tissues and secretory organs. In this study, we assessed how the enhancement of two targeting and cell-penetrating peptides to the developed Ce6 composition would affect the accumulation of PS in healthy and tumor tissue. Modification of the Ce6 phospholipid composition with peptides made it possible to increase the PS level in the tumor tissue by almost 1.5 times compared to free Ce6 1 h after PS administration. At the same time, the PS level in the tumor tissue was two times higher than in the liver and kidneys 1 h after administration of phospholipid NP with peptides. This confirms our previous in vitro findings [24]. Most likely, this effect is due to the selective effect of a peptide with the NGR motif on the protein aminopeptidase N/CD13 expressed on the surface of HT-1080 cells, which was initially proven using Western blot. It was also shown that chlorin e6 is almost completely eliminated by the liver and kidneys, with the maximum accumulation occurring 30 min after the administration of either of the two phospholipid compositions. Kataoka et al. reported that conjugation of Ce6 with glucose or mannose increased antitumor activity, as well as tumor targeting compared to conventional PSs. The authors attribute this action to the Warburg effect, a phenomenon in which cancer cells uptake higher levels of glucose than normal cells [38]. In our case, most likely, the unique properties of the phospholipid system, as well as the effect of increased permeability and retention in the tumor tissue, resulted in greater accumulation in the tumor tissue. The extremely small size of nanoparticles (up to 40 nm) [24] facilitates their passage through various physiological barriers. Additional enhancement of the composition with a targeting peptide with the NGR motif responsible for binding to the CD13 receptor protein makes the system targeted, and the arginine-containing cell-penetrating peptide, due to guanidinium groups of Arg that ensure internalization into the cell, helps to transport Ce6 inside the cell, according to [39]. In [17], the asparagine–glycine–arginine (NGR) motif in thermosensitive liposomes (TSL) containing a conjugate of CPP with doxorubicin was also used as a target fragment, which confirmed the specific ability and improved intracellular delivery of liposomes to HT-1080 cells in vitro. TSL significantly inhibited tumor growth in nude mice with HT-1080 xenograft tumors. However, it should be noted that our particles with Ce6 and peptides are two times smaller than TSL (40 nm versus 90 nm).

Thus, enhancing the PS with a delivery system containing a targeting component, in our case, the NGR peptide specific for aminopeptidase N (APN; CD13) and also cell-penetrating (R7), contributed to an increase in the Ce6 selectivity to tumor cells, which is undoubtedly a key element in the treatment of cancer. Studies of the use of aminopeptidase N (CD13) as a signaling molecule of the progression of many cancers have great potential. However, there are still many unresolved and unexplored issues regarding its expression both on the surface of tumor and healthy cells [28]. Our work is aimed at using this receptor as a target for the PS Ce6 phospholipid delivery system with two targeting and cell-penetrating peptides. Moreover, it has been reliably shown that the modification of the phospholipid composition with two types of peptides improves the properties of the previously developed Ce6 phospholipid composition, namely, the selectivity of its action in vitro and in vivo. However, it should be noted that there are some unresolved issues related to the mechanism of action of the developed system, which, in turn, will further increase the efficacy of PDT in the treatment of cancer.

## 5. Conclusions

Confocal microscopy used to assess the intracellular distribution of Ce6 revealed partially mitochondrial localization after incubation with the peptide-modified Ce6 composition (NPh-Ce6-NGR-R7). The arginine-containing cell-penetrating peptide may have played a decisive role in this distribution. The tumor cell death assessment demonstrated that the cells died by apoptosis, most likely of the mitochondrial type, which confirmed the mitochondrial localization of the PS. A study of the in vivo distribution of the PS showed a higher accumulation of the photosensitizer in the tumor after administration of the two-peptide Ce6 composition (NPh-Ce6-NGR-R7). Thus, the proposed chlorin e6 composition simultaneously modified with two peptides was shown to be a promising tool for PDT. Further evaluation of the use of phospholipid delivery systems with targeting peptides may provide new opportunities for agents with poor water solubility and target delivery of such compounds to tumor cells. Ce6 can be considered a promising agent for the treatment of epithelial cancers. Combinatory treatment studies could be suggested as the next step of non-clinical evaluation to determine clinical perspectives of the agent.

## Data Availability

The data are available on reasonable request from the corresponding author.

## Supplementary Materials

The following supporting information can be downloaded at: https://www.mdpi.com/article/10.3390/pharmaceutics14102224/s1, Figure S1: correlation coefficient of colocalization using MitoTracker for Ce6; Figure S2: correlation coefficient of colocalization using MitoTracker for NPh-Ce6; Figure S3: correlation coefficient of colocalization using MitoTracker for NPh-Ce6-NGR-R7; Figure S4: 3D cell reconstruction the HT-1080 for Ce6; Figure S5: 3D cell reconstruction the HT-1080 for NPh-Ce6; Figure S6: 3D cell reconstruction the HT-1080 for NPh-Ce6-NGR-R7; Video S1: Cell death for Ce6 in HT-1080; Video S2: Cell death for NPh-Ce6 in HT-1080; Video S3: Cell death for NPh-Ce6-NGR-R7 in HT-1080.
