# Supplementary figures and images for: Chlorin e6 Phospholipid Delivery System Featuring APN/CD13 Targeting Peptides: Cell Death Pathways, Cell Localization, In Vivo Biodistribution
Source: Pharmaceutics. 2022 Oct 18;14(10):2224. doi: 10.3390/pharmaceutics14102224 (PMC9610949; doi:10.3390/pharmaceutics14102224)

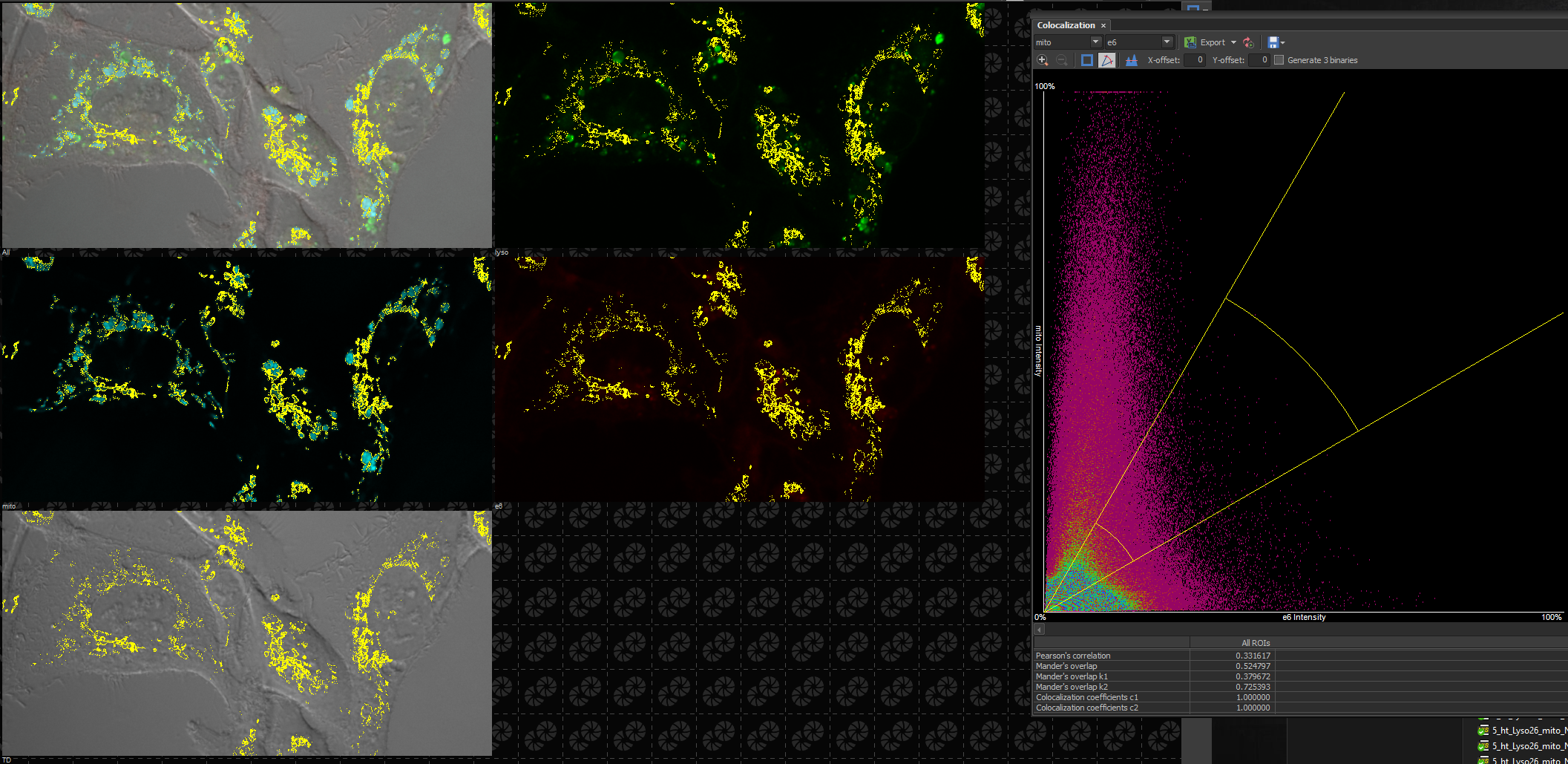

Supplement: Supplementary file 1 [file pharmaceutics-14-02224-s001.zip › Figure S1. Correlation coefficient of colocalization using MitoTracker for Ce6_1.png]

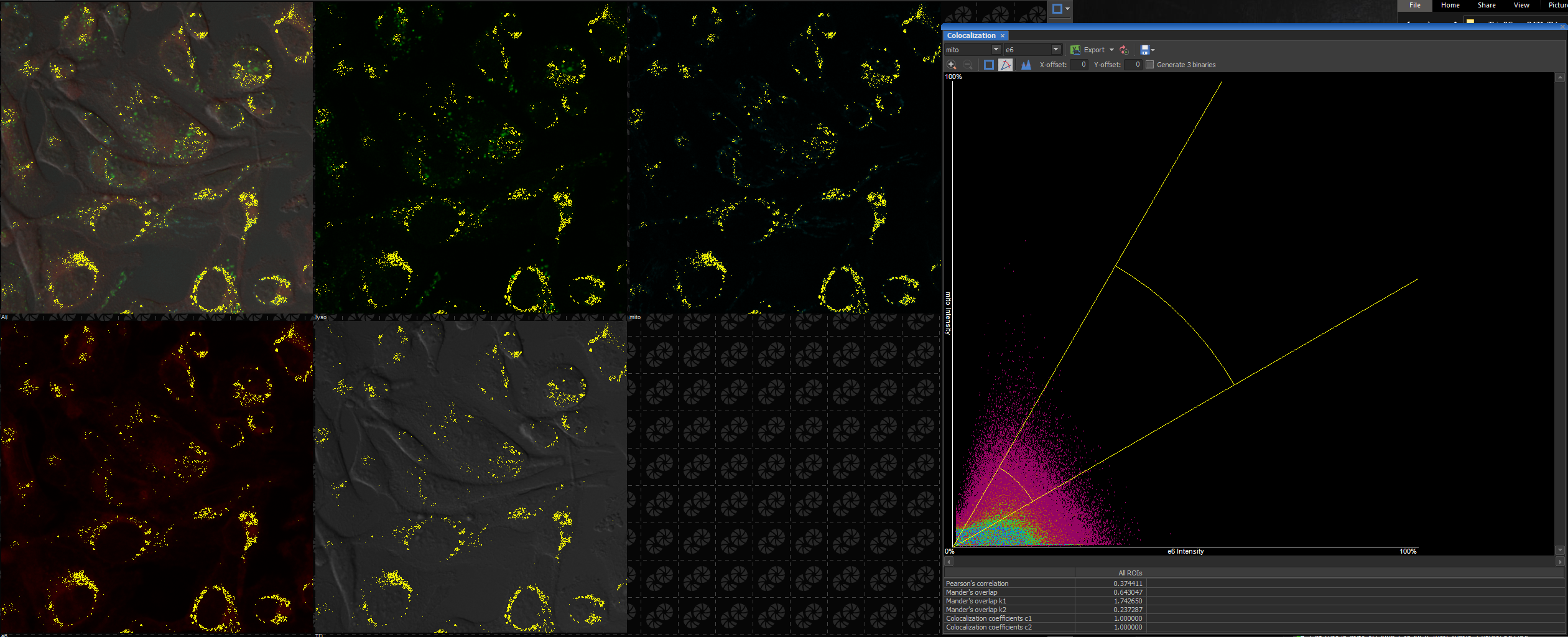

Supplement: Supplementary file 1 [file pharmaceutics-14-02224-s001.zip › Figure S2. Correlation coefficient of colocalization using MitoTracker for NPh-Ce6.png]

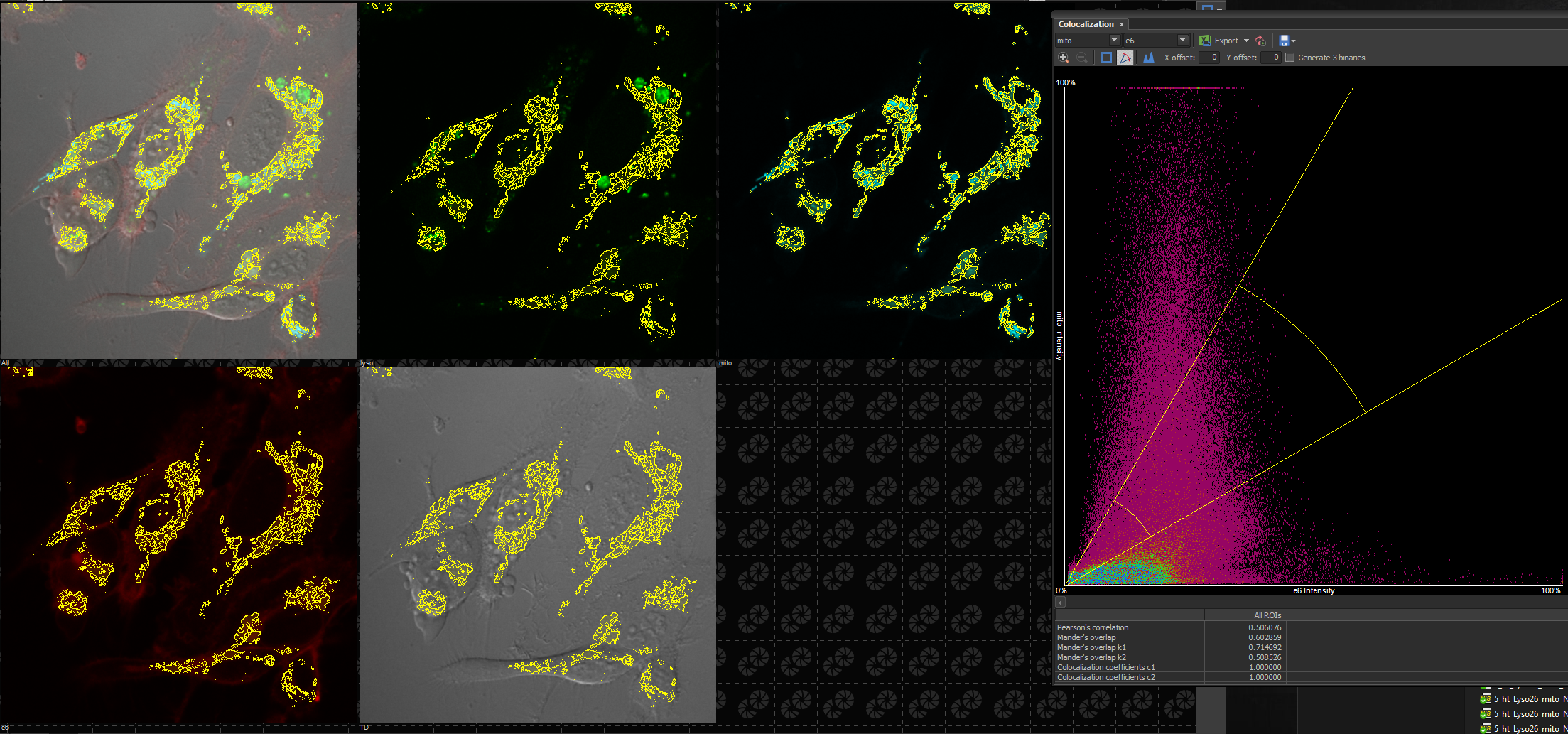

Supplement: Supplementary file 1 [file pharmaceutics-14-02224-s001.zip › Figure S3. Correlation coefficient of colocalization using MitoTracker for NPh-Ce6-NGR-R7.png]

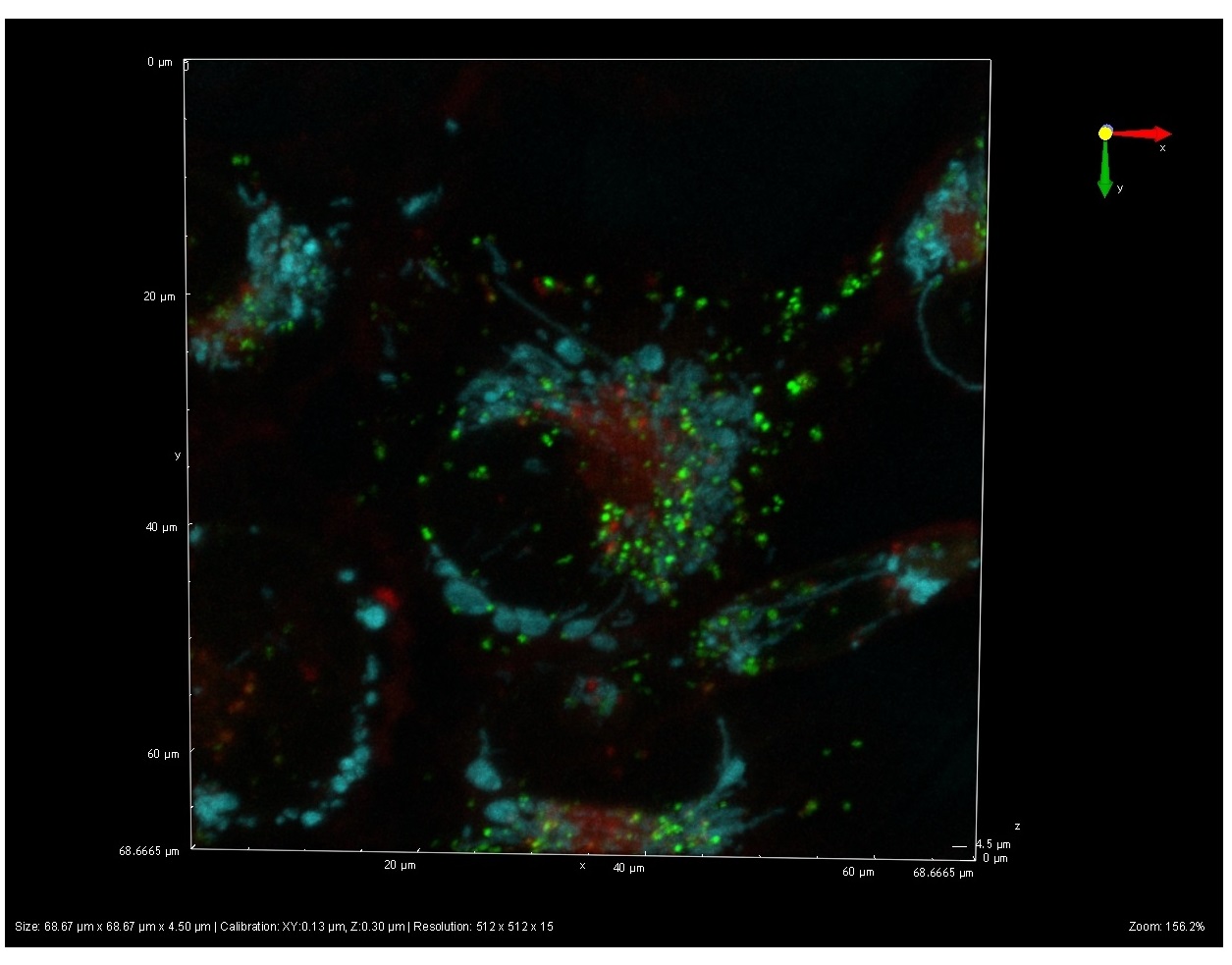

Supplement: Supplementary file 1 [file pharmaceutics-14-02224-s001.zip › Figure S4. 3D cell reconstruction the HT-1080 for Ce6.jpg]

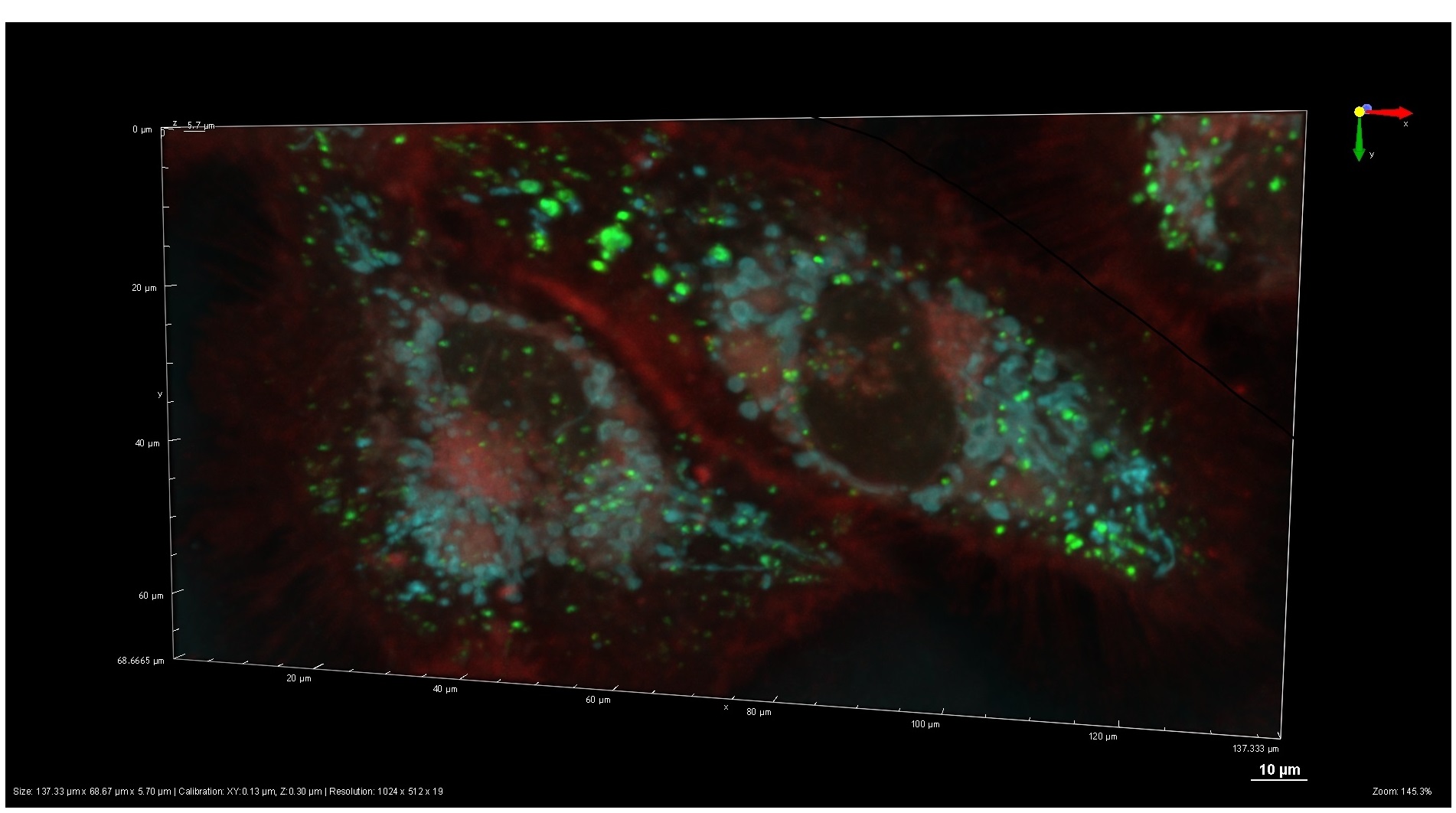

Supplement: Supplementary file 1 [file pharmaceutics-14-02224-s001.zip › Figure S5. 3D cell reconstruction the HT-1080 for NPh-Ce6.jpg]

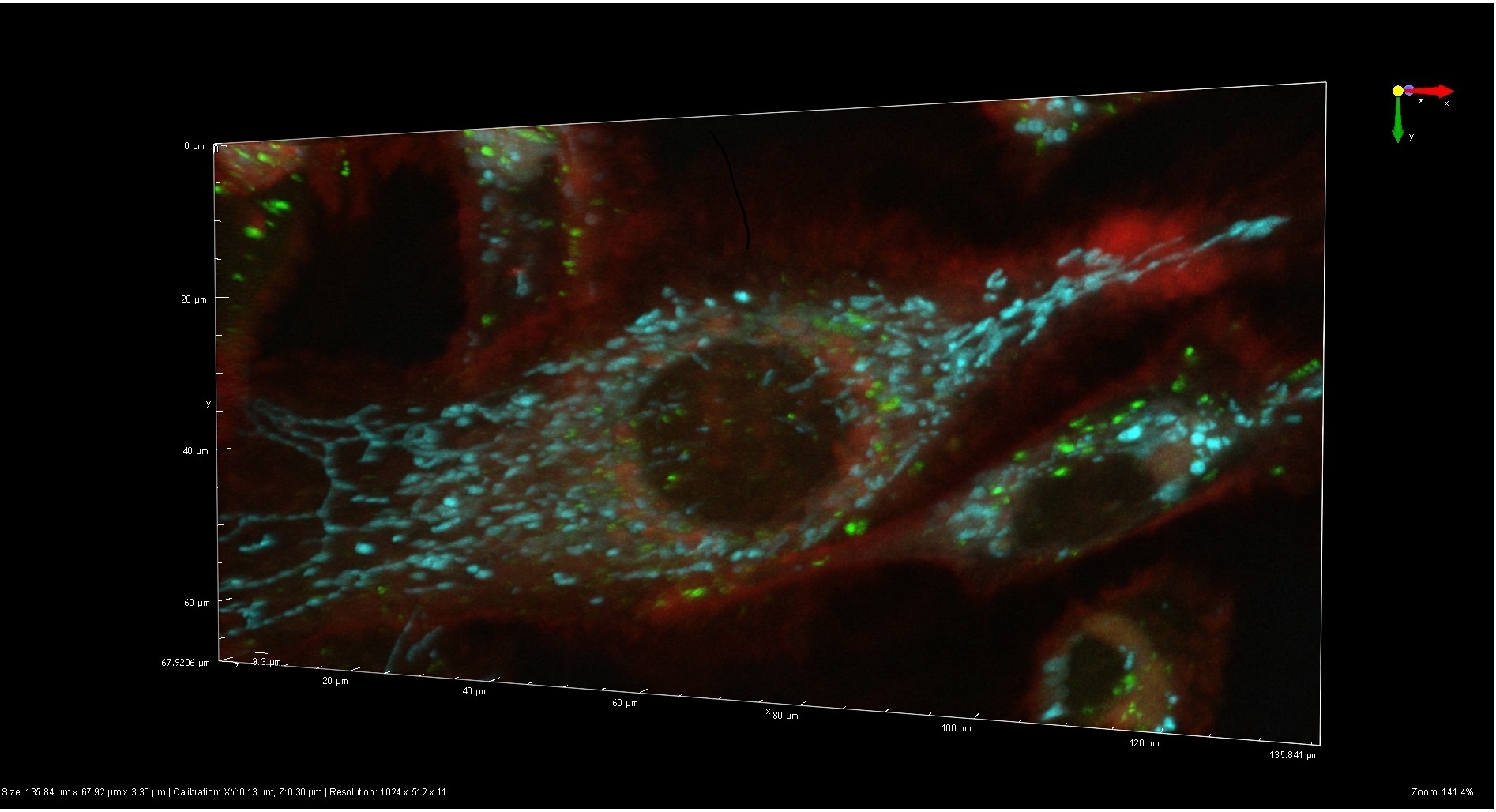

Supplement: Supplementary file 1 [file pharmaceutics-14-02224-s001.zip › Figure S6. 3D cell reconstruction the HT-1080 for NPh-Ce6-NGR-R7.jpg]
